# Supplementary material for: Regulating strain in perovskite thin films through charge-transport layers
Source: Nat Commun. 2020 Mar 23;11:1514. doi: 10.1038/s41467-020-15338-1 (PMC7090003; doi:10.1038/s41467-020-15338-1)
Supplement: Supplementary file 1 — Supplementary Information [file 41467_2020_15338_MOESM1_ESM.pdf]

# Supplementary Information for

## Regulating strain in perovskite thin films through charge-transport layers

Ding-Jiang Xue,[1,2†] Yi Hou,[1†] Shun-Chang Liu,[2] Mingyang Wei,[1] Bin Chen,[1] Ziru Huang,[1] Zongbao Li,[2,4] Bin Sun,[1] Andrew H. Proppe,[1,3] Yitong Dong,[1] Makhsud I. Saidaminov,[1] Shana O. Kelley,[3,5] Jin-Song Hu,[2] and Edward H. Sargent[1*]

[1]Department of Electrical and Computer Engineering, University of Toronto, Toronto, Ontario M5S 1A4, Canada

[2]Beijing National Laboratory for Molecular Sciences (BNLMS), CAS Key Laboratory of Molecular Nanostructure and Nanotechnology, Institute of Chemistry, Chinese Academy of Sciences, Beijing 100190, China

[3]Department of Chemistry, University of Toronto, Toronto, Ontario M5S 3G4, Canada

[4]National Engineering Research Center for Advanced Polymer Processing Technology, Zhengzhou University, Zhengzhou, 450002, China

[5]Deparetment of Pharmaceutical Sciences, Leslie Dan Faculty of Pharmacy, University of Toronto, Toronto, Ontario M5S 3M2, Canada

[†]These authors contributed equally to this work: Ding-Jiang Xue, Yi Hou

[*]e-mail: ted.sargent@utoronto.ca

This file includes Supplementary Figures 1 to 18.

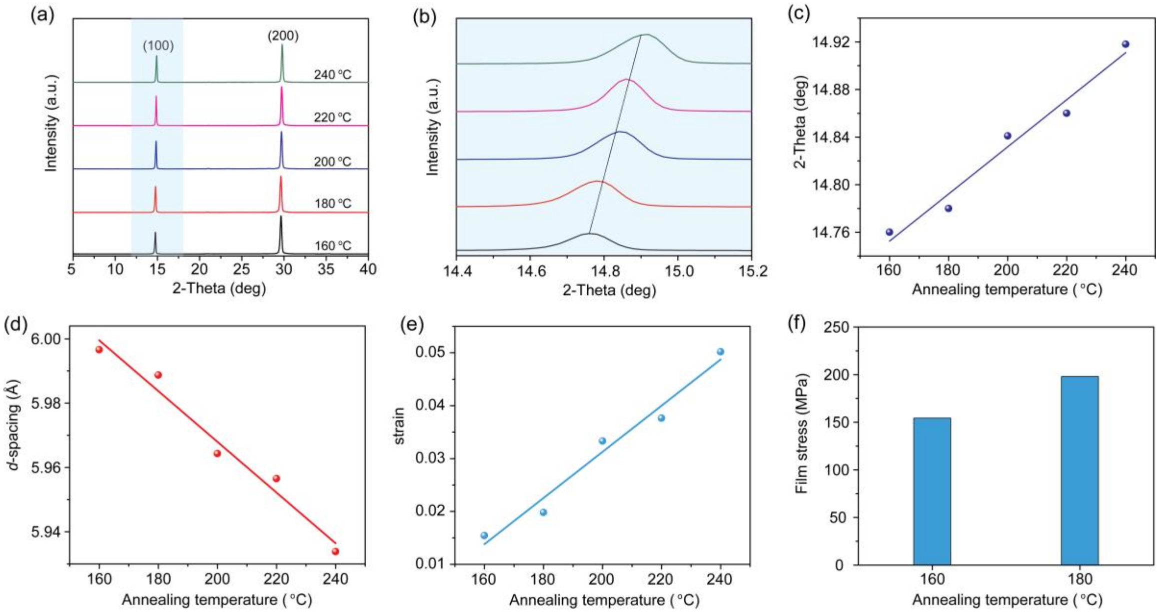


**Supplementary Figure 1. Characterizations of strain in perovskite films formed at different temperatures.** (a) XRD patterns of $CsPbI_2Br$ film formed at different temperatures from 160 to 240 $^oC$. (b) Magnified (100) diffraction peaks in the region indicated by the blue. (c) Measured (100) peak positions of $CsPbI_2Br$ films formed at different temperatures. (d) Measured (100) *d*-spacing of $CsPbI_2Br$ films formed at different temperatures. (e) Measured strain in $CsPbI_2Br$ films formed at different temperatures. (f) Measured stress in $CsPbI_2Br$ films formed at 160 $^oC$ and 180 $^oC$.

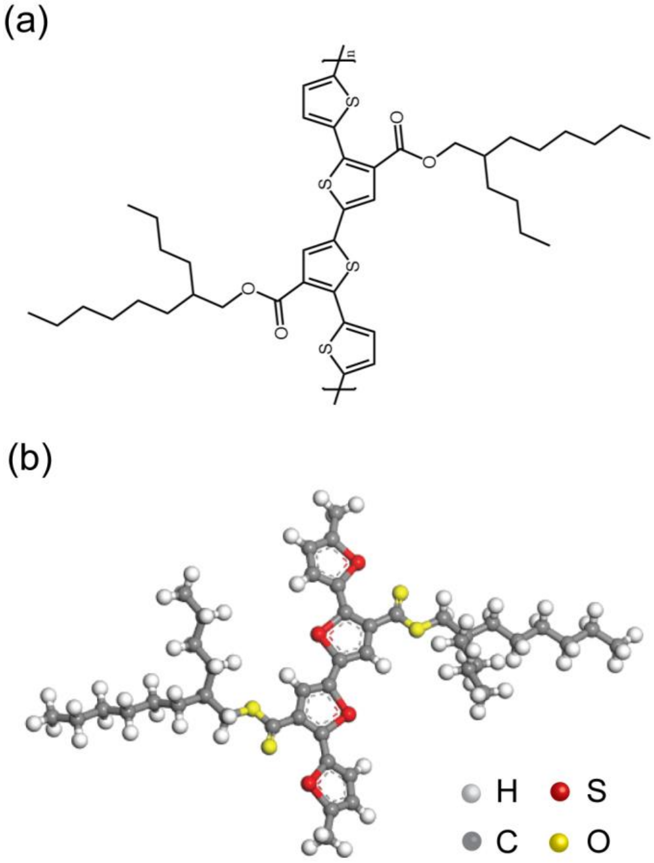


**Supplementary Figure 2.** (a) Chemical structure of PDCBT. (b) 3D structure of PDCBT.

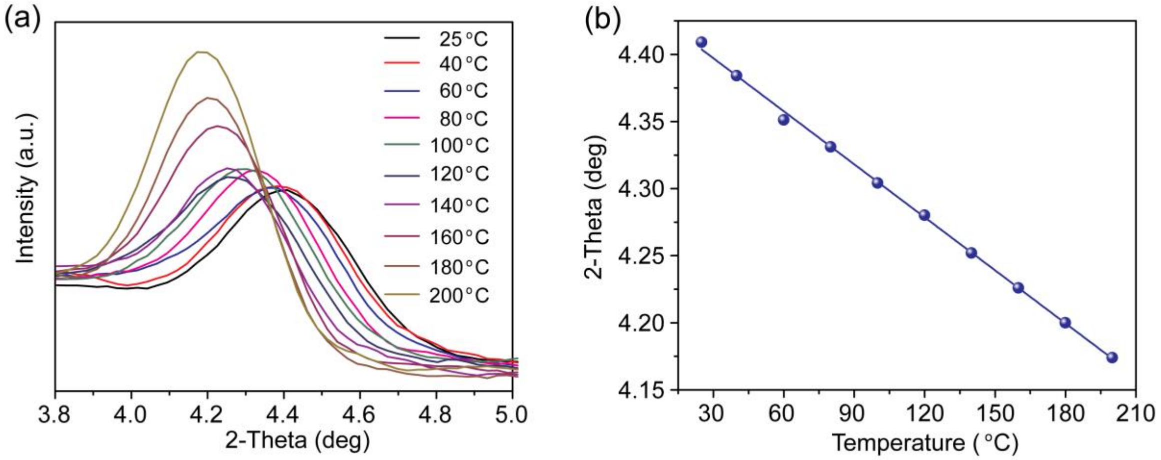


**Supplementary Figure 3. Characterization of thermal expansion coefficient of PDCBT.** (a) Temperature-dependent XRD patterns of PDCBT from 25 to 200 $^{o}$C. (b) Measured (100) peak position of PDCBT film at different temperatures.

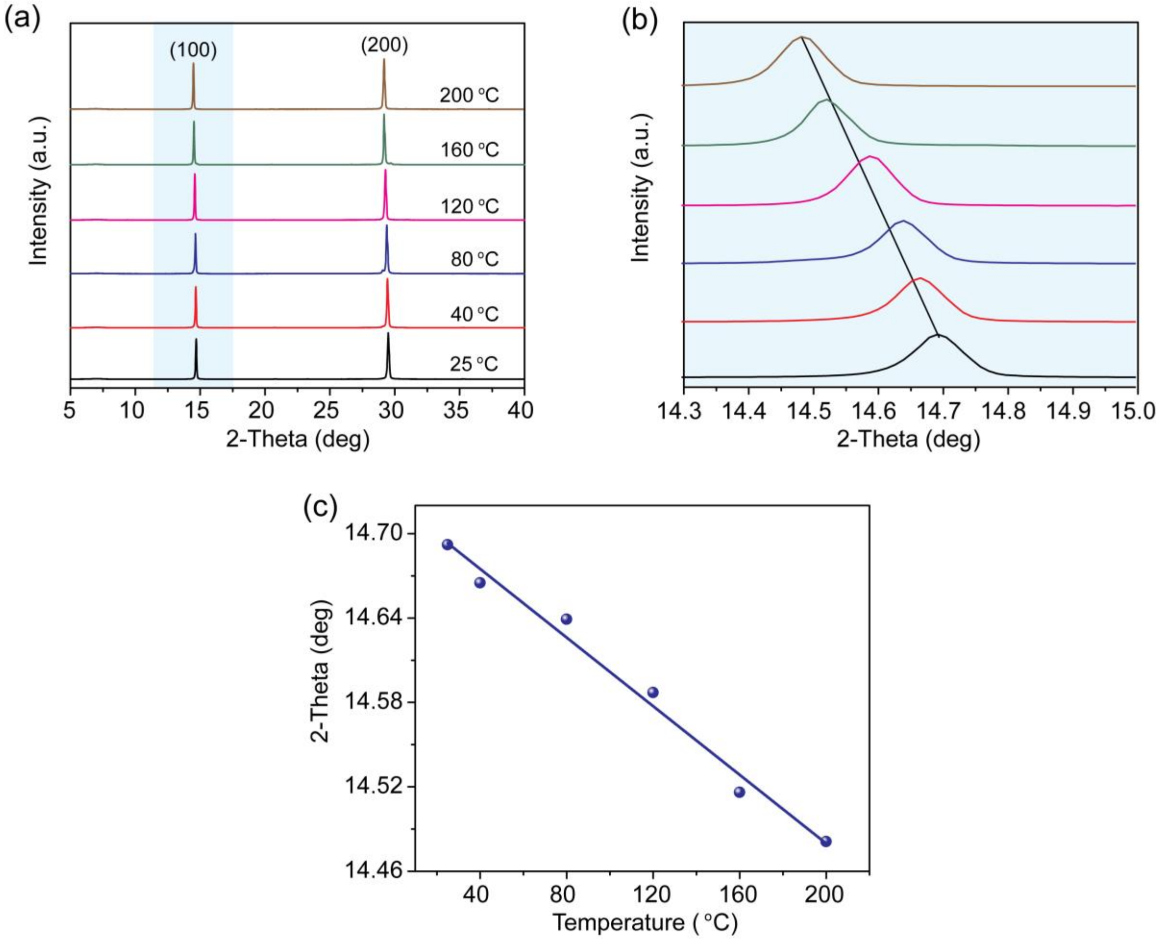


**Supplementary Figure 4. Characterization of thermal expansion coefficient of $CsPbI_2Br$.** (a) Temperature-dependent XRD patterns of $CsPbI_2Br$ film from 25 to 200 °C. (b) Magnified (100) diffraction peaks in the region indicated by the blue. (c) Measured (100) peak position of $CsPbI_2Br$ film at different temperatures.

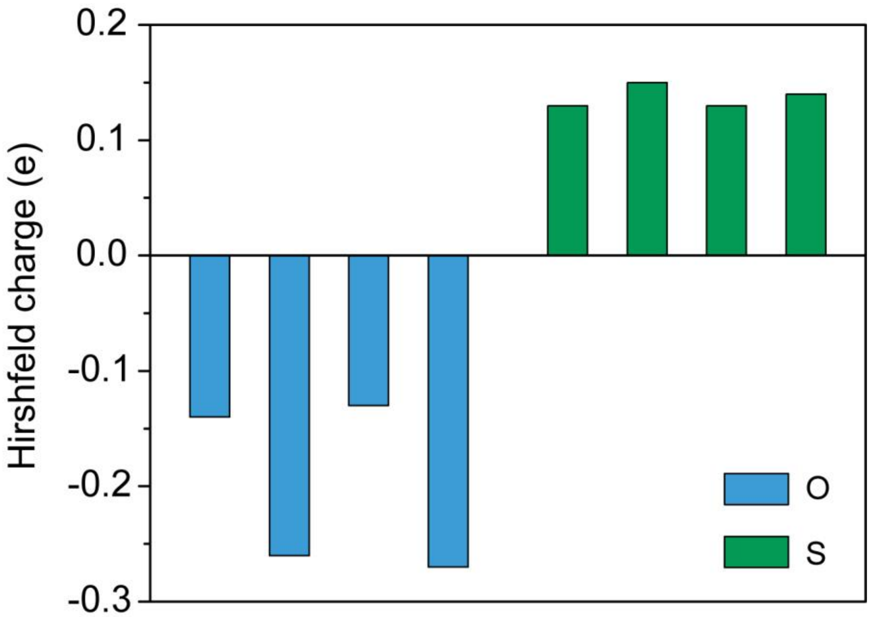


**Supplementary Figure 5.** Calculated Hirshfeld charges of O and S in PDCBT.

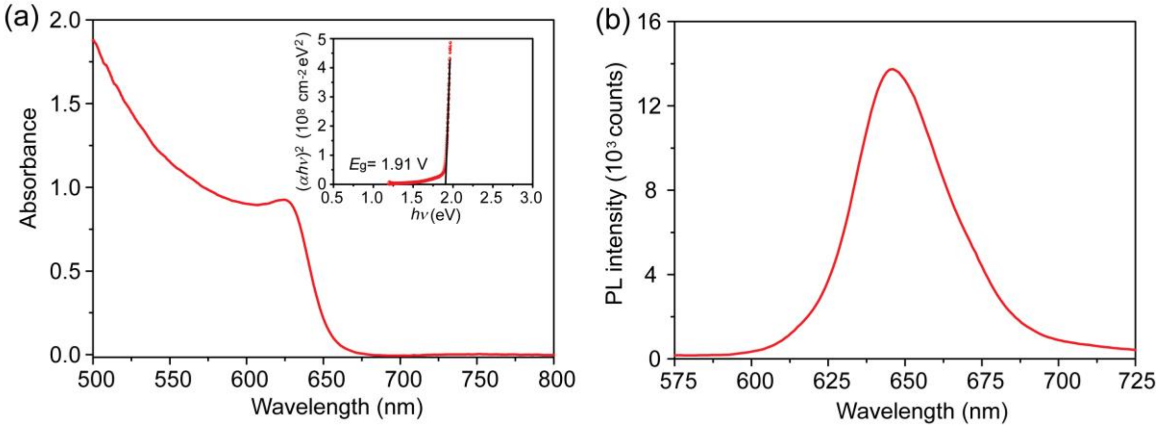


**Supplementary Figure 6. Optical characterizations of $CsPbI_2Br$ film formed at 160 ºC.** (a) Absorption spectrum of $CsPbI_2Br$ film. Inset: Tauc plot for $CsPbI_2Br$ film to determine the bandgap of $CsPbI_2Br$. (b) PL spectrum of $CsPbI_2Br$ film.

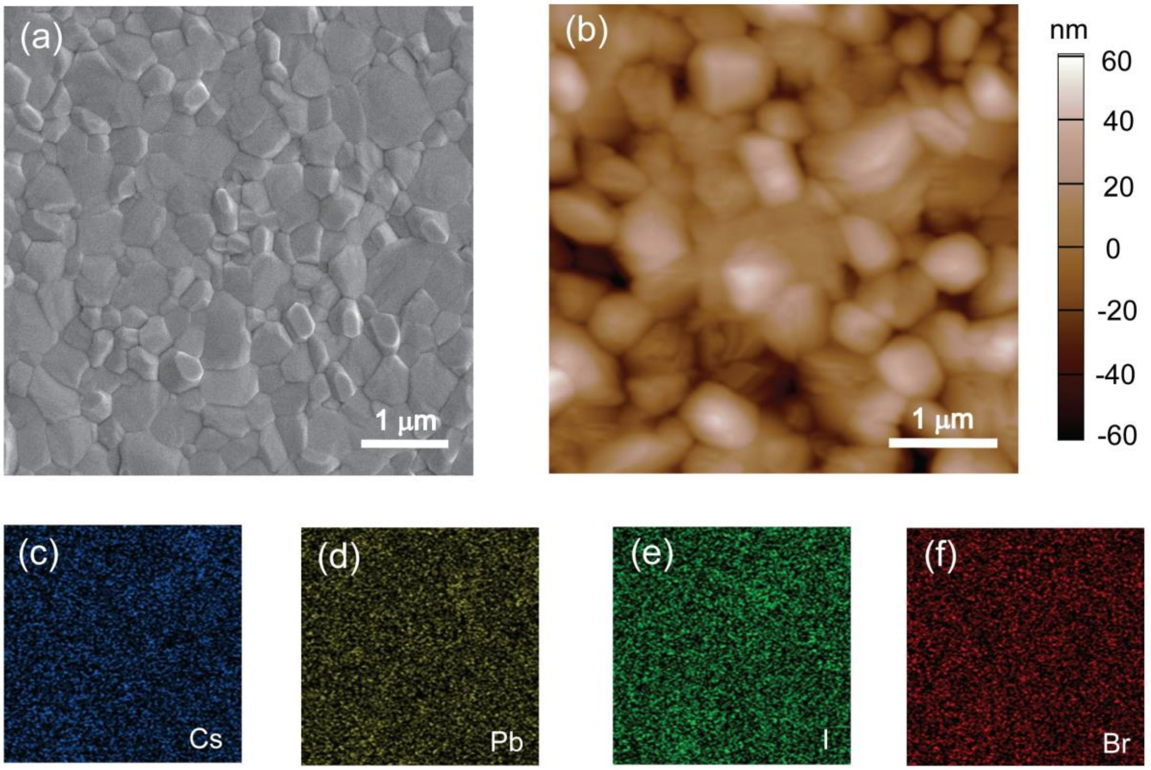


**Supplementary Figure 7. Material characterizations of $CsPbI_2Br$ film formed at 160 $^oC$.** (a) SEM top-view image of $CsPbI_2Br$ film. (b) AFM image of $CsPbI_2Br$ film. EDS elemental maps of (c) Cs, (d) Pb, (e) I, and (f) Br.

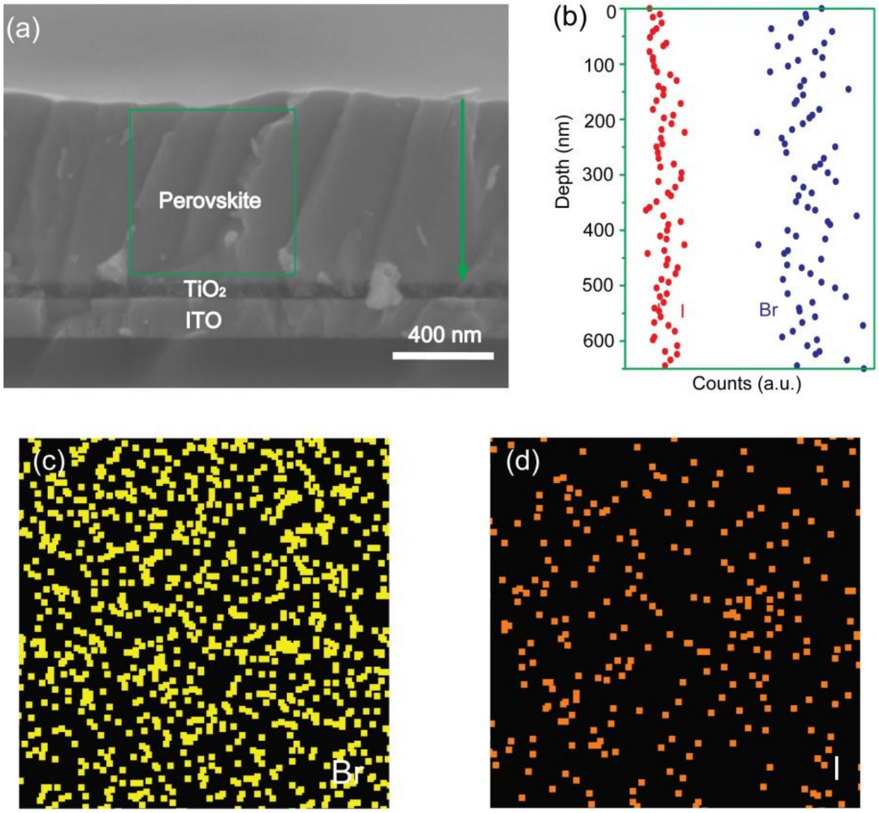


**Supplementary Figure 8. Material characterizations of $CsPbI_2Br$ film formed at 160 $^{o}$C.** (a) SEM top-view image of $CsPbI_2Br$ film. (b) AFM image of $CsPbI_2Br$ film. EDS elemental maps of (c) Cs, (d) Pb, (e) I, and (f) Br.

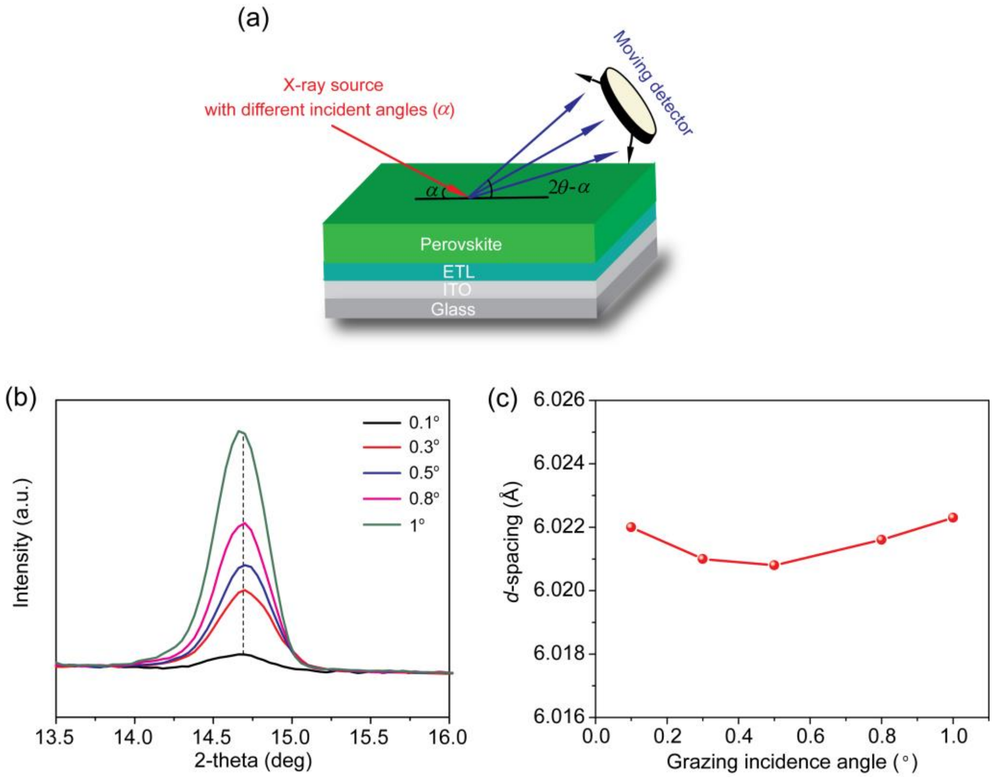


**Supplementary Figure 9. Characterization of the strain distribution in $CsPbI_2Br$ film formed at 160 $^oC$.** (a) Scheme of the grazing incidence XRD (GIXRD) measurement. (b) GIXRD patterns of $CsPbI_2Br$ film as a function of incident angle $\alpha$. (c) $d$-spacing values obtained from $CsPbI_2Br$ (100) plane as a function of $\alpha$. By using incident angles varying from 0.1$^o$ to 1$^o$, we are able to reveal the structural differences in the perovskite layer at a theoretical probing depth of ~30 nm to 3000 nm[1,2].

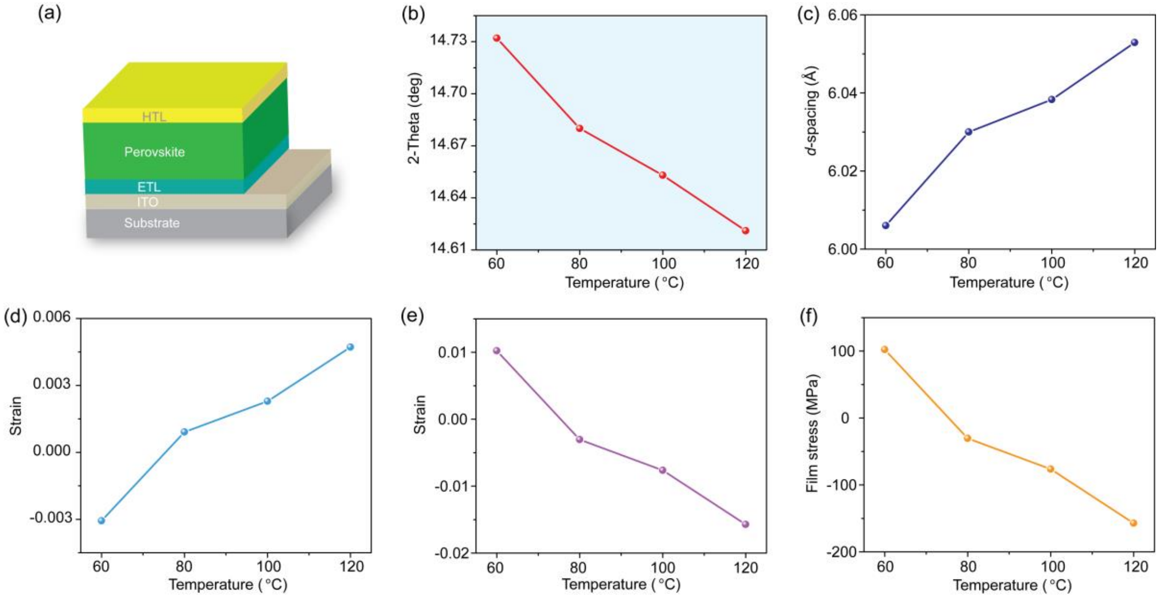


**Supplementary Figure 10. Characterizations of strain in $CsPbI_2Br$/PDCBT films fabricated at different PDCBT spin-coating temperatures.** (a) Schematic configuration of PSCs. (b) Measured (100) peak positions of $CsPbI_2Br$ as a function of PDCBT processing temperature. (c) Measured (100) *d*-spacing of $CsPbI_2Br$ as a function of PDCBT processing temperature. (d) Measured strain in $CsPbI_2Br$ films in the direction perpendicular to the substrate as a function of PDCBT processing temperature. (e) Measured strain in $CsPbI_2Br$ films in the direction horizontal to the substrate as a function of PDCBT processing temperature. (f) Measured stress in $CsPbI_2Br$ films in the direction horizontal to the substrate as a function of PDCBT processing temperature.

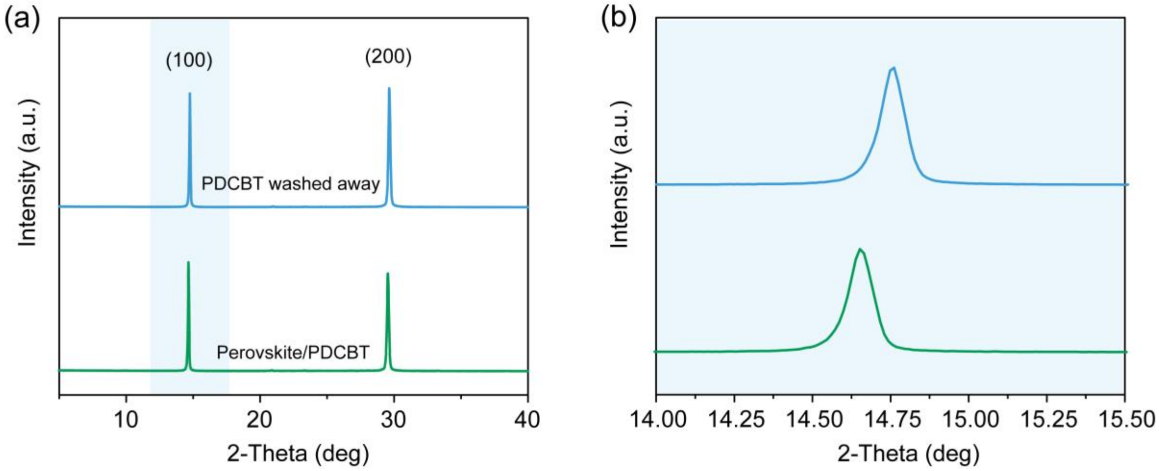


**Supplementary Figure 11. Characterization of strain in perovskite films.** (a) XRD patterns of perovskite/PDCBT films fabricated with a PDCBT spin-coating temperature of 120 $^{o}$C, and perovskite films after the PDCBT layer is washed away. (b) Magnified (100) diffraction peaks in the highlighted (blue) region.

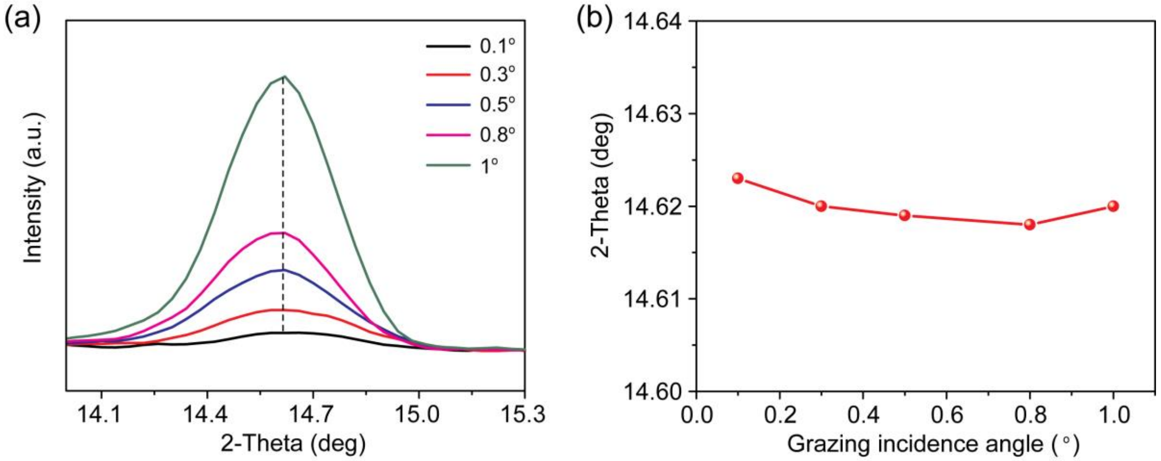


**Supplementary Figure 12. Characterization of the strain distribution in $CsPbI_2Br$ film when spin-coating the PDCBT at 120 ºC.** (a) GIXRD patterns of $CsPbI_2Br$ film as a function of incident angle $\alpha$. (b) peak positions obtained from $CsPbI_2Br$ (100) plane as a function of $\alpha$.

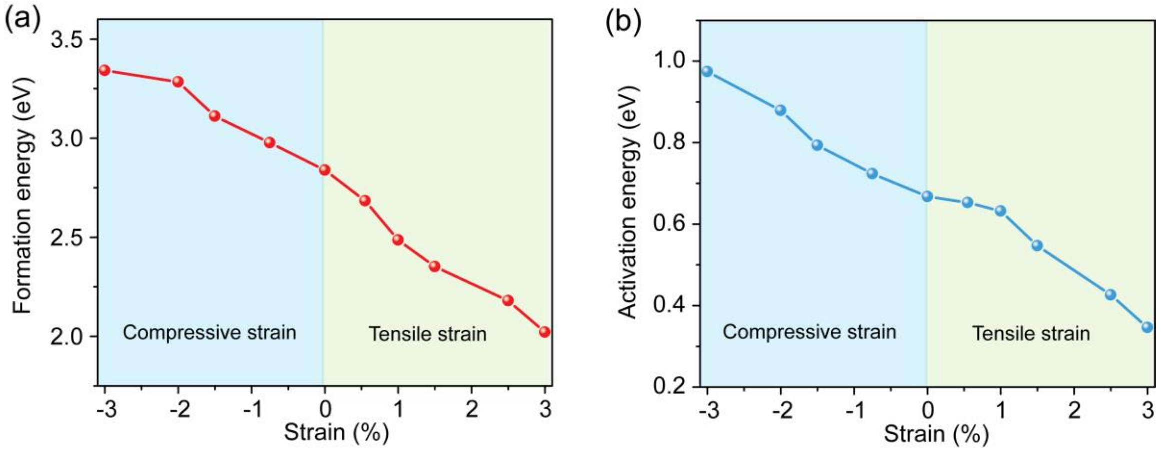


**Supplementary Figure 13. Halide vacancy and ion migration from DFT calculations.** (a) Calculated strain-dependent formation energies of halide vacancies. (b) Calculated strain-dependent activation energies for the vacancy-assisted migration of halide ions.

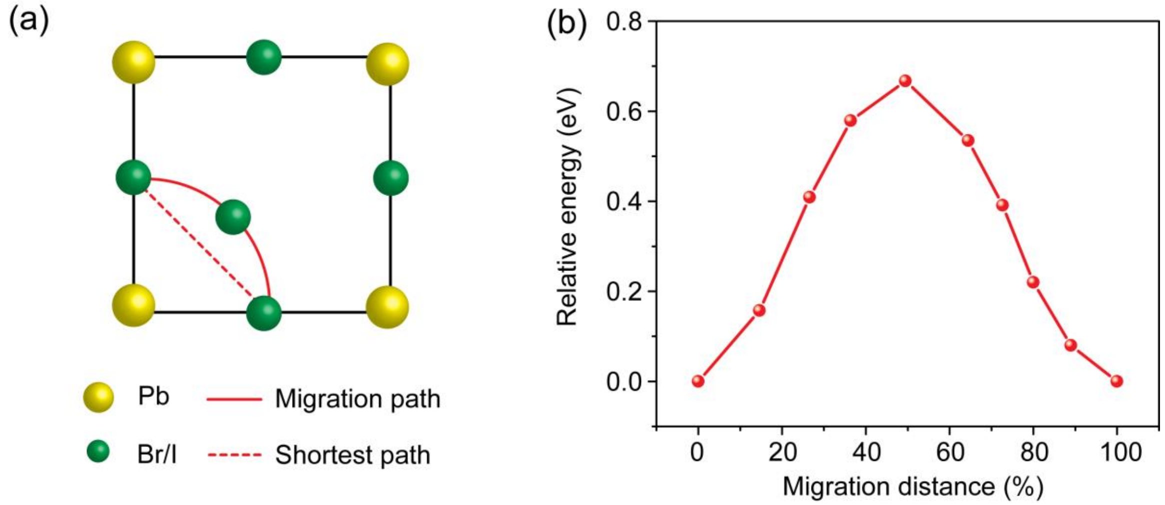


**Supplementary Figure 14. Halide ion vacancy migration from DFT calculations.** (a) Scheme of vacancy-assisted migration of halide ions. (b) Corresponding relative energy as a function of migration distance.

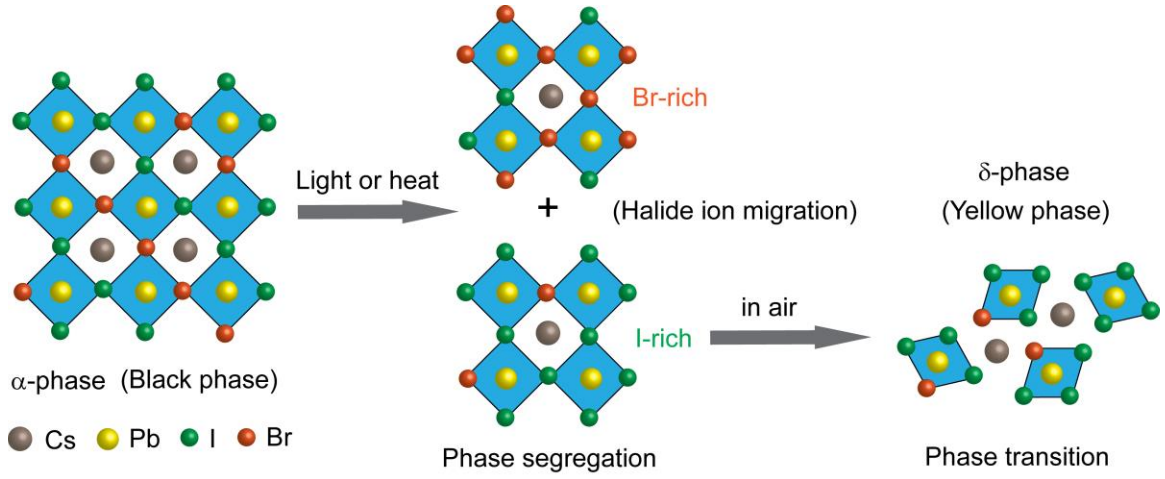


**Supplementary Figure 15.** Schematic of phase segregation and transition in $CsPbI_2Br$.

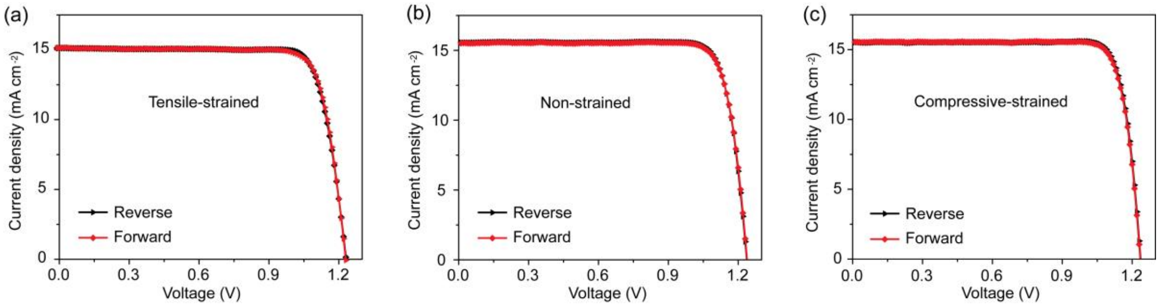


**Supplementary Figure 16.** *J-V* curves of (a) tensile-strain, (b) non-strain and (c) compressive-strain PSCs with reverse and forward scan.

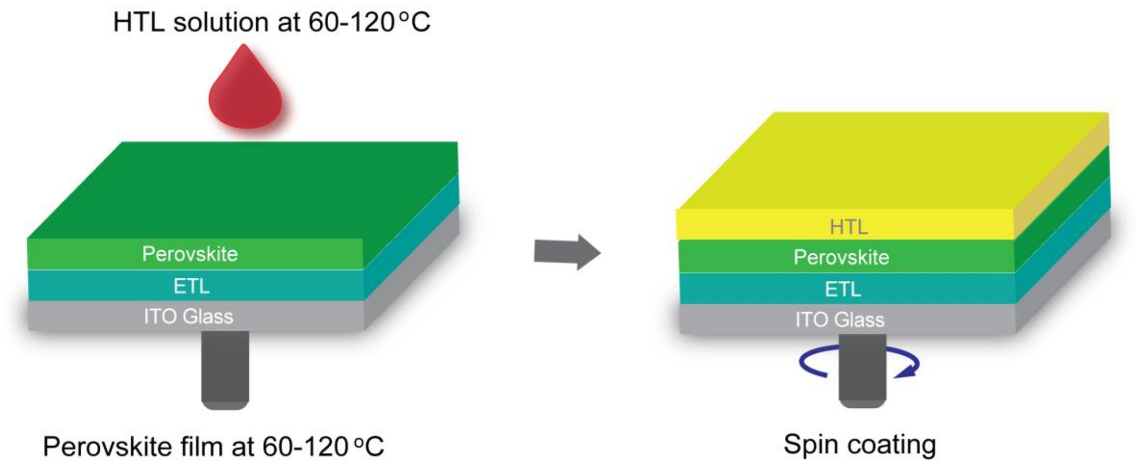


**Supplementary Figure 17.** Hot-casting scheme for the deposition of HTL.

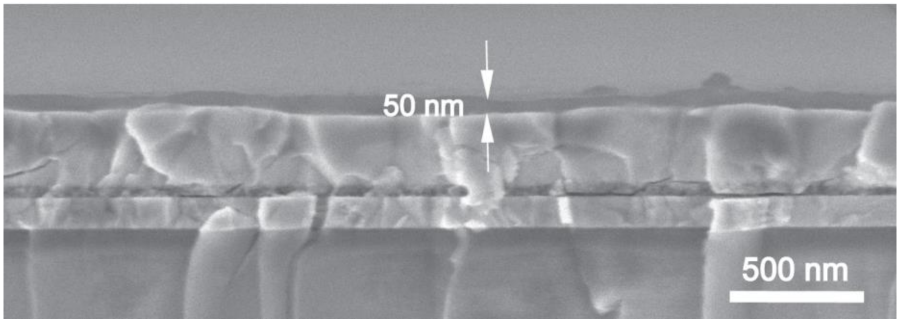


**Supplementary Figure 18.** SEM top-view image of $CsPbI_2Br$/PDCBT film.

**Supplementary References**


1. Choi, Y. C. & Seok, S. I. Efficient $Sb_2Se_3$-sensitized solar cells via single-step deposition of $Sb_2S_3$ using S/Sb-ratio-controlled $SbCl_3$-thiourea complex solution. *Adv. Funct. Mater.* **25**, 2892-2898 (2015).
2. Yang, Y. et al. Enhanced crystalline phase purity of $CH_3NH_3PbI_{3-x}Cl_x$ film for high-efficiency hysteresis-free perovskite solar cells. *ACS Appl. Mater. Interfaces* **9**, 23141-23151 (2017).
